# Bibliography

**Published:** 1842-03

**Authors:** 


*Bibliography.*—We have several dental works which it was our intention to have noticed in the present number of the Journal, but are prevented from doing so for want of room; we shall endeavour however, to attend to them in our next.

